# A protocol for developing an evaluation framework for an academic and private-sector partnership to assess the impact of major food and beverage companies’ investments in community health in the United States

**DOI:** 10.1186/s40608-015-0066-0

**Published:** 2015-09-24

**Authors:** Terry T-K Huang, Emily Ferris, Rachel Crossley, Michelle Guillermin, Sergio Costa, John Cawley

**Affiliations:** School of Public Health, City University of New York, New York, NY USA; Broadwaters Advisory Services Ltd., Tunbridge Wells, UK; Healthy Weight Commitment Foundation, Washington, DC USA; Department of Policy Analysis and Management, Cornell University, Ithaca, NY USA

## Abstract

Public health leaders increasingly recognize the importance of multi-sector partnerships and systems approaches to address obesity. Public-private partnerships (PPP), which are joint ventures between government agencies and private sector entities, may help facilitate this process, but need to be delivered through comprehensive, transparent frameworks to maximize potential benefits and minimize potential risks for all partners. The City University of New York (CUNY) School of Public Health and the Healthy Weight Commitment Foundation (HWCF) propose to engage in a unique academic-private-sector research partnership to evaluate the impact and effectiveness of the food and beverage industry’s investment in obesity and hunger prevention and reduction through community-level healthful eating and active living programs. The CUNY-HWCF academic-private partnership protocol described here incorporates best practices from the literature on PPP into the partnership’s design. The CUNY-HWCF partnership design demonstrates how established guidelines for partnership components will actively incorporate and promote the principles of successful PPPs identified in various research papers. These identified principles of successful PPP, including mutuality (a reciprocal relationship between entities), and equality among partners, recognition of partners’ unique strengths and roles, alignment of resources and expertise toward a common cause, and coordination and delegation of responsibilities, will be embedded throughout the design of governance, management, funding, intellectual property and accountability structures. The CUNY-HWCF partnership responds to the call for increased multi-sector work in obesity prevention and control. This framework aims to promote transparency and the shared benefits of complementary expertise while minimizing shared risks and conflicts of interest. This framework serves as a template for future academic-private research partnerships.

## Correspondence

### Background

Despite significant investments in obesity prevention, the prevalence of obesity remains persistently high. In the United States, 68.5 % of adults are overweight or obese and 31.7 % of children and adolescents ages 2-19 years are overweight or obese [1].^,^ Globally, in 2014, 39 % of adults were affected by overweight or obesity [2]. In recognition of the complexity of the obesity problem, leading public health organizations and UN system organizations including the World Health Organization (WHO) have called for increased partnerships and multi-stakeholder approaches among other policy approaches to address obesity and diet-related diseases [3]. The Institute of Medicine further stresses the importance of systems change to accelerate progress in obesity prevention and to ensure the synergistic involvement of multiple sectors, stakeholders and strategies [4]. Food and beverage companies are part of this complex system and play a substantial role in shaping the food landscape. Public health leaders increasingly recognize the need to engage industry leadership and action to achieve a systems approach to obesity and food insecurity prevention, although best practices for engagement remain underdeveloped. The Obesity Society recently issued a position statement supporting and encouraging science-industry collaborations where transparency and scientific rigor are maintained [5, 6]. A growing body of research, including a recent paper identifying 12 principles to building Public-Private Partnerships (PPPs), synthesizes and promotes the underlying principles or best practices of successful PPPs [7–10].

Public-private partnership (PPP) refers to a diverse range of collaborations between private and public-sector entities with varying types of participants, governance, management, legal status, policy-setting, contributions and operations [11]. Objectives vary by partnerships and can include product development or distribution, strengthening direct services, research, policy development, or education [11]. Partnership between the scientific or academic sector and the food and beverage industry are a specific type of PPP and is the focus of this paper. Such partnership, if properly designed, can help facilitate a multi-stakeholder approach and contribute to achieving the goals of increasing the public’s consumption of healthful foods and reducing food insecurity [7]. In addition, some companies, recognizing potential alignment between this work and business goals, are interested in playing a role in shifting social values towards health-promoting lifestyles. However, understanding the dimensions of a successful partnership and establishing principles and guidelines to ensure accountable partnerships are key to helping partners manage conflict and navigate challenges. A review of existing research on PPP guidelines and principles identified several congruent elements of successful partnerships including: mutuality and equality among partners; accountability and transparency; recognition of partners’ unique strengths and roles; and alignment of joint assets to support a well-defined, common mission and objectives [7, 9]. To help ensure transparency and manage potential conflicts associated with PPP, partners can create comprehensive partnership protocols and written policies covering terms, outcomes, benefits, institutional processes, and guidelines for conflicts of interest [7, 8, 10]. To our knowledge, there have yet to be published case studies translating this research on successful principles of PPP into practice specifically for community-based interventions that support obesity prevention and healthy lifestyles.

### CUNY-HWCF research partnership

The City University of New York (CUNY) School of Public Health and the Healthy Weight Commitment Foundation (HWCF), a CEO-led coalition with over 275 member organizations, are engaging in a research partnership to assess the food and beverage industry’s investment in community-based healthful eating, active living and healthy weight initiatives [12]. HWCF was formed in 2009 to help food and beverage companies and other organizations with similar goals work together to address childhood obesity [12]. HWCF currently includes 17 corporate members and close to 300 associate members. Since its inception, HWCF has engaged in several domestic campaigns and PPPs, including a calorie-reduction pledge from member companies who removed 6.4 trillion calories from the marketplace and Together Counts, an initiative which provides families and schools with curriculum and tools to support active and healthy living [12]. This research partnership follows these previous HWCF PPP.

Members of the Grocery Manufacturers Association (GMA), to which many HWCF members also belong, invested approximately $100 million in food access, nutrition education and physical fitness programs from 2010–2013 and there is significant interest in evaluating the impact of this investment [13]. The CUNY-HWCF partnership’s evaluation will focus initially on the diverse obesity and food access programs within the United States undertaken by HWCF corporate members in the food and beverage sector.

CUNY and HWCF propose to engage in a unique research partnership which differs from both more traditional PPPs and academic-private collaborations. PPPs such as this one involving academic partners have some strengths relative to PPPs with typical public-sector partners. Private-sector partners may be more inclined to share data with academic partners than other types of public partners knowing the information will not be used against them in regulatory or judicial settings. In addition, academic partners can provide technical assistance and further partnerships’ ability to generate new knowledge. The CUNY-HWCF research partnership also differs from previous academic-private collaborations. Whereas academic teams often independently evaluate private-sector partners or activities, in this research partnership the academic team will work more directly with companies through HWCF to develop an evaluation framework to assess the companies themselves. This interactive approach will help leverage the business and research expertise from each partner and will ensure that the output is both relevant and useful to the industry and public health community.

The partnership product will benchmark industry investment in and involvement with healthful eating and active living programs, with the goal of helping companies make smarter investments and create greater community health impact in the long run. In essence, the product will function as a monitoring system and the methodology, industry-level results and resulting public discourse constitute one form of industry accountability [14].

In this paper, we describe the CUNY-HWCF research partnership protocol, building on the evolution of PPP in obesity prevention and control and the current call for increased multi-stakeholder approaches [3, 4]. The protocol synthesizes previous research on principles for building successful PPP and then uses these principles as a set of best practices to inform the partnership’s design and to maximize benefits and minimize risks. As interest in PPP in obesity prevention and control continues to grow and evolve, this study protocol can serve as a tool for future academic-private partnerships.

### Methods/Design

The research partnership design provides a framework for how the CUNY-HWCF initiative will function and builds on research on the principles of successful PPP, proposed best practices and lessons learned from PPPs [7–10]. The partnership also draws from existing benchmarking systems such as the Access to Nutrition Index and the Global Reporting Initiative [15, 16]. This project, however, differs from these established benchmarking systems as it focuses solely on evaluating community-level healthful eating and active living programs. Because each PPP has unique characteristics and objectives, this partnership framework incorporates specific, relevant elements from several articles addressing the principles of successful PPPs [7–10]. The partnership design creates clear, comprehensive guidelines for different project elements including governance, management, and accountability structures, in order to ensure transparency and manage potential conflicts of interest. The project structures are designed to build trust and goodwill among partners and promote elements of successful PPPs identified by previous research such as mutuality and equality, accountability and transparency, recognition of partners’ unique strengths and roles, alignment of resources and expertise toward a common cause and coordination and delegation of responsibilities.

#### Governance

An established, explicit governance structure recognizes each partner’s unique roles and supports the coordination and delegation of responsibilities in research partnerships. A review of lessons learned from existing global public-private health partnerships identified effective, transparent governance as one strategy to improve PPP success and efficacy [8]. Clear governance structures are intended to help partners manage potential conflicts and maintain accountability. Governance structures may include outside organizations or groups to provide expertise and hold partners accountable to the agreed upon protocol.

**Fig 1:**
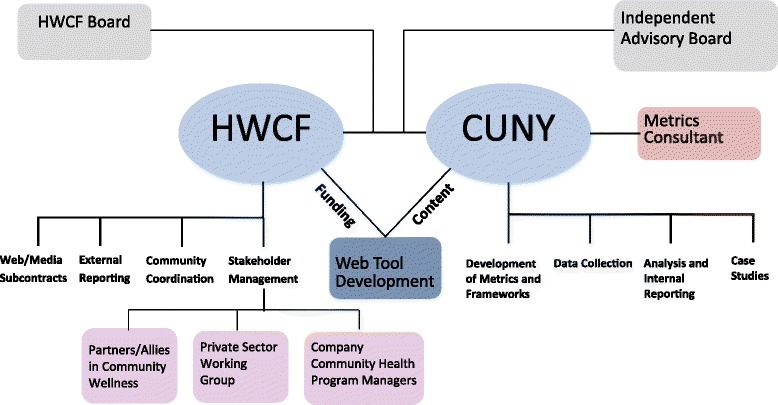
Governance Structure. Figure 1 illustrates CUNY and HWCF’s specific governance and leadership roles within the CUNY-HWCF academic-private research partnership. An established governance structure recognizes partners’ unique roles and supports the delegation and coordination of responsibilities. The Independent Advisory Board, comprised of outside public health and management science experts, provides advice to support the partnership’s work and hold partners accountable to the agreed upon research framework

In the context of the CUNY-HWCF research partnership, CUNY and HWCF have specific governance and leadership roles and an Independent Advisory Board (IAB) will provide additional guidance, as shown in Fig. 1. Using its research and evaluation expertise, CUNY will develop metrics and evaluation frameworks, collect and analyze data, and develop case studies. HWCF will coordinate and work with participating companies through a private sector working group and through a group of company-level community health program managers. HWCF will also ally with organizations that share an interest in wellness and in benchmarking company’s community investment and programming.

The IAB serves as an additional governance structure in the CUNY-HWCF partnership. With diverse public health and industry expertise, IAB members will provide advice and insights to enhance the partnership’s work. By monitoring the partnership’s progress, the board will also help hold partners accountable to the agreed upon research framework.

#### Roles and responsibilities

Beyond governance structures, establishing clear, comprehensive roles and responsibilities for all partners is one of 12 proposed principles to developing effective PPP [9]. Distinct roles and responsibilities recognize partners’ unique strengths and areas of expertise, promote transparency and mitigate potential conflicts of interest. Clear guidelines within the partnership protocol further align partners’ assets in support of their common goal and help build mutual trust and respect [9].

In keeping with this approach to roles and responsibilities, CUNY and HWCF’s distinct roles in the partnership are based on their unique strengths and optimal alignment of joint resources. With research and evaluation expertise, CUNY will develop the research methodology and assessment tools. The research methodology will include three components: an inventory framework; an evaluation framework; and a community-level assessment. An inventory framework will catalogue the different types and components of industry investment in community-based healthful eating and active living initiatives. An evaluation framework will assess the management, quality and impact of programming at the company and program levels. The CUNY team will develop a unique evaluation framework for this project, but will draw from existing evaluation frameworks and industry benchmarking systems such as the Collective Impact Model and the London Benchmarking Group [17, 18]. CUNY will also develop a community-level impact assessment to evaluate a subset of company programs in order to capture industry investment at this level and will share relevant results with each sampled community. In addition to developing the evaluation framework, CUNY will analyze all collected data to understand the quality and impact of the industry’s investment and create confidential scorecards for each participating company to communicate the evaluation findings. Using its industry expertise, HWCF will contribute to the design of the inventory and evaluation frameworks to ensure their relevance and usefulness to the industry. HWCF member companies may provide input during the design process, but HWCF and CUNY will be responsible for the final design. In addition, HWCF will oversee communication and coordination between the research partner and participating companies. HWCF will communicate to the participating companies a “best practices” document based upon the evaluation framework. HWCF will coordinate participating companies and publicly issue aggregate industry reports to share key findings with interested and affected stakeholders including community leaders, non-governmental organizations and the media. The aggregate industry reports will include quantitative data analysis by the CUNY team, as well as qualitative case studies curated jointly by the CUNY and HWCF communications teams. Such reports will include findings on strengths as well as weaknesses in current activities, and opportunities for improvement.

#### Funding flows

Understanding and disclosing funding flows is an important component of PPPs. Disclosure helps ensure transparent financial relationships. The design of funding structures may also help mitigate potential conflicts of interests.

The funding structure of the CUNY-HWCF project seeks to minimize potential risks of PPPs. Most significantly, HWCF functions as a holding entity of funds from multiple private sources. The pooled funds will be subcontracted to various research partners for technical assistance. Because of this structure, no single contributor of funds will dominate the decision-making process.

#### Intellectual property and communication of data

As academic-research partnerships intend to generate new ideas and data, intellectual property and data communication protocols will be developed early on. This further ensures that partners understand their distinct roles and contributions to the project and the additional transparency helps mitigate potential risks.

HWCF and CUNY will share intellectual property rights over the project’s data. The partnership will only report on data based on pre-established frameworks. Companies will have access to their own data, but company reports of findings or data analysis will need to use and reference the research framework, to ensure consistency with reports from the CUNY-HWCF partnership. HWCF and CUNY will co-brand and co-author communication of data, as appropriate.

#### Accountability

Research on PPP has also identified the importance of explicitly addressing accountability mechanisms [8]. This further establishes trust and mutuality among partners and increases project credibility by holding partners to their agreed upon roles. Accountability structures address how partners will hold each other accountable as well as how partnerships will be held accountable to other stakeholders and outside organizations. Accountability mechanisms also provide guidelines for addressing potential conflicts.

The CUNY-HWCF partnership includes several structural mechanisms and established guidelines which hold all partners accountable to their roles and responsibilities. Partner accountability mechanisms include pre-set milestones to keep partners on track and to support partner’s established roles and responsibilities. Regular stakeholder consultations help ensure that the frameworks and end products will be relevant and useful.

The IAB will provide feedback and guidance to improve the partnership products and will help hold members responsible to the agreed research framework. The IAB will also mitigate potential limitations of the partnership’s reliance on mutual accountability. Effectively assessing their own performance, governance or accountability mechanisms may be challenging for partnerships which rely heavily on mutual accountability [19]. Independent groups, such as the IAB, may be able to better monitor or critique a partnership in an unbiased and productive manner.

The CUNY-HWCF partnership framework includes some guidelines to address potential conflicts. Should one party misrepresent the results, the other party can publicly and contractually dissolve the partnership and dissociate itself from any communication by which the misrepresentation is made. If any user directly or indirectly associated with either partnership entity misrepresents the results, the partners agree to persuade the user to retract or rectify the misrepresentation or in the case of refusal, to publicly correct such misrepresentation.

In addition to taking the account and holding the account, an established four-step accountability framework includes two other important dimensions of accountability: sharing the account and responding to the account [19]. Sharing the account fosters transparency and dialogue among stakeholders [19]. This research partnership framework serves as an initial communication to share accountability expectations and initiate dialogue among stakeholders regarding the CUNY-HWCF partnership. The partnership will also provide a publicly available industry-level report of the assessment’s findings (both positive and negative) to further share evidence and actively engage the media, impacted communities and other interested stakeholders.

Responding to the account ensures that partnerships monitor and improve performance and accountability mechanisms [19]. The CUNY research team is actively engaged in ongoing dialogue with HWCF as well as CEOs and representatives of participating companies. One of the by-products of this partnership is the opening of a forum where new ideas are being shared, in hopes of broadening the willingness, interest, and knowledge base among all parties with regard to PPPs in the field of nutrition and obesity. In addition, the IAB will provide the CUNY-HWCF partnership with feedback to help monitor performance and, if needed, will help strengthen accountability structures. In addition, the industry score cards will help companies monitor their progress and identify ways to improve their performance in obesity prevention and food access work.

### Discussion

Due to the complexity of the obesity problem facing the U.S. and many other countries, and the growing call for multi-sectoral partnerships, PPPs, including academic-research partnerships, are likely to be increasingly important vehicles to address obesity. This academic-private partnership protocol seeks to incorporate the existing research on successful PPPs into a practical framework to guide the CUNY-HWCF partnership and future academic-private partnerships. The design of the partnership’s governance and accountability structures demonstrates how the CUNY-HWCF partnership will incorporate the principles of effective PPP identified in existing research [7–10].

Balancing industry and public health interests is an important component of a research partnership. Open communication and transparent project development can help identify where industry and public health priorities overlap and diverge [7]. Identifying anticipated benefits may result in the alignment of resources towards common goals, while identifying potential risks furthers transparency and mitigates potential conflicts.

#### Shared benefits

The CUNY-HWCF research partnership includes several shared benefits for both partners. Many PPPs benefit from complementary expertise and pairing human resources capacity [8]. The resources and assets provided by both partners will further their common goals. The academic team’s evaluation, research and public health expertise combined with HWCF’s industry knowledge and support will lead to a highly translatable, practical product. This product will guide industry investment in programs addressing healthful eating and active living and maximize the investment’s impact. The evaluation process further seeks to foster a culture of inquiry and inspire companies to share knowledge and lessons learned with each other and other interested stakeholders. By establishing common metrics and evaluating the actual impact of company’s healthful eating and active living strategies and programs, the research partnership has the ability to develop evidence-based solutions and potentially transform industry’s engagement with obesity prevention and control. This research partnership model will create a new platform for PPPs to create social good.

Potential benefits could extend beyond the research partnership. The project’s methodology may complement the Access to Nutrition Index (ATNI) which ranks and assesses global manufacturers on nutrition-related commitments, practices and disclosure [15]. Engaging allied organizations with similar goals could lead to the additional benefit of connecting the research partnership to a larger movement. The research partnership’s process and product contributes to the broader thinking around corporate social responsibility and investment in obesity prevention and control.

#### Shared risks and potential conflicts of interest

The CUNY-HWCF partnership also includes shared and individual risks for partners. The framework seeks to minimize these risks and potential conflicts of interest through establishing clear, transparent guidelines.

The complexity of the project poses one shared risk. Evaluating the industry’s investment may require a greater volume of information and a different set of analytic tools than anticipated. The evaluation will use a staged approach, collecting data at different levels. This will include an inventory of general information and a more detailed evaluative approach at the company, program and single-community levels.

Provision of data may present an additional shared risk. With the development of an inventory tool, the research partner may need to provide technical assistance or one-on-one consultation with company representatives if they encounter data entry challenges. Company data may exist in different formats, adding another layer of complexity. Acknowledging the limitations of self-reported data and the potential for misreporting, under reporting or over reporting, data quality is a potential concern. False or whitewashed data would reduce the credibility and effectiveness of the product, creating a risk for both partners. Companies’ voluntary participation and interest in maximizing their investments’ effectiveness should mitigate this potential risk. A random audit will be conducted for a subset of companies that will further protect against this risk.

The sustainability of funding may also pose a potential risk to the partnership. It may be challenging to articulate the relevance and the importance of the output of this work to companies to maintain their continued support.

The undue impact of corporate influence on study design and outcomes could pose as a conflict of interest and negatively affect the project’s credibility. To increase transparency and mitigate this potential risk, the research partnership framework provides specific guidelines for how HWCF and participating companies will contribute to and influence the partnership’s process and product. There will be regular consultations with companies so the leadership and staff both understand what is being proposed and why. Both CUNY and HWCF will help companies understand the value of ensuring rigor, integrity, and credibility in the scientific process. In addition, explicit funding and governance structures, public disclosure of research frameworks and findings, and shared credit and responsibility over publications all serve to mitigate the risk of conflicts of interest. The publication of the current protocol also affirms both HWCF and CUNY’s joint acknowledgement and ownership of this risk, serving to further incentivize both partners to work within the agreed upon framework.

The IAB will mitigate the risk of corporate influence or conflicts of interest on the project’s credibility by monitoring for evidence of undue corporate influence or emerging conflicts of interest throughout the partnership’s duration. Board members will represent specific stakeholder groups and provide diverse perspectives and critiques. IAB members will review partnership frameworks and methodologies and ensure adherence to good practices throughout the process.

#### Conclusion

The CUNY-HWCF research partnership responds to the need for multi-sector approaches to address the complexity of the obesity problem. By drawing from existing research on principles of successful PPP and proposed PPP best practices, the research partnership framework plans to maximize partnership benefits while minimizing any potential risks. Explicit funding and governance structures as well as clearly defined roles and responsibilities will mitigate potential conflicts of interest. Mutuality and equality will be developed through an understanding of how partners’ unique assets and expertise will complement each other and further their shared goals. The agreed upon roles and responsibilities will further support the coordination and delegation of responsibilities. The research partnership framework seeks to make the process transparent and will facilitate the partnership’s work. In addition, the protocol established here may serve as a useful tool for future academic-private PPPs.

Through the CUNY-HWCF research partnership, we seek to learn a great deal about industry’s investment in obesity and hunger prevention and reduction in the United States. We may learn more about the distribution of funds, the types of programs being implemented, and how approaches differ across companies. In addition, we may learn to what extent this work will lead to sustained engagement with PPPs. This partnership and evaluation process represents an important step towards improving the design and strategy of industry programs. The CUNY-HWCF partnership aims to serve as a vehicle to change how different sectors communicate and to inspire innovation.
